# Results for introduction of a new hand hygiene program in the ICU

**DOI:** 10.1186/cc11990

**Published:** 2013-03-19

**Authors:** P Vos, A Harts-Laurijsen, R Snoeren, R Ramnarain, JA Van Oers

**Affiliations:** 1 St Elisabeth Hospital, Tilburg, the Netherlands

## Introduction

Although hand hygiene is known as a core element in prevention of healthcare-associated infections, compliance to its program is not high. With a new campaign we tried to enlarge awareness on hand hygiene.

## Methods

We performed an experimental, before and after, study design on our 30-bed, mixed medical-surgical ICU. We conducted a baseline evaluation during 3 days in May 2012 including direct observation of hand hygiene compliance by control nurses and hand cultures of 50 healthcare workers (HCW). Based on the WHO Guidelines on Hand Hygiene in Health Care [1], cleaning of hands with alcohol-based hand rubs (Sterillium) was prescribed before touching a patient and before aseptic procedures, after body fluid exposure risk and after touching a patient and touching his/her surroundings. Promotion of the hand hygiene program consisted of lectures and web-based self-learning, posters located near points of care and verbal reminders by control nurses. New observations of hand hygiene by control nurses during 3 days and hand cultures of 50 healthcare providers were performed in September 2012. Consumption of alcohol-based hand rub (product volume use per patient-days) was used as a surrogate marker of hand hygiene over time. The difference in hand hygiene compliance during the two periods was examined using a chi-squared test. Differences in hand cultures were examined using a Student's *t *test. Time trends in the consumption of alcohol-based hand rub were examined using linear correlation. *P <*0.05 was considered statistically significant. The study was approved by the institutional Ethics Review Board.

## Results

During the survey, in May 158 opportunities to observe hand hygiene were presented and 286 in September. Overall compliance improved from 34.2% (54/158) to 51% (146/286), *X*^2 ^= 11.7 (*P <*0.001). In May, 50 HCW had a mean of 63.20 ± 39.37 colony-forming units (CFU) on their hands compared with 43.0 ± 40.19 CFU on the hands of 50 HCW in September (*P *= 0.024). We also observed an initial increased use of alcohol-based hand rubs from 21 ml per patient-day in May to a maximum 72 ml per patient-day in June, but a decline to 44 ml per patient-day in September, Pearson correlation coefficient = 0.31 (*P *= 0.61).

## Conclusion

Implementation of a new hand hygiene program at our ICU resulted in improved hand hygiene compliance and less CFU on the hands of HCW. There was no significant increased use of alcohol-based hand rubs over time. The results indicate that constant awareness is vital for success.
